# An Integrated Approach Using GA-XGBoost and GMM-RegGAN for Marine Corrosion Prediction Under Small Sample Size

**DOI:** 10.3390/ma18163760

**Published:** 2025-08-11

**Authors:** Qian Chen, Yikun Cai, Yuqin Zhu, Haodi Ji, Xiaobing Ma, Han Wang

**Affiliations:** 1 School of Reliability and Systems Engineering, Beihang University, Beijing 100191, China; qianchen@buaa.edu.cn (Q.C.); jhd2021@buaa.edu.cn (H.J.); 2 School of Aeronautics and Astronautics, Sichuan University, Chengdu 610065, China; caiyikun@scu.edu.cn; 3 Southwest Institute of Technology and Engineering, Chongqing 400039, China; 12234117@zju.edu.cn; 4 Ocean College, Zhejiang University, Hangzhou 316021, China

**Keywords:** marine steel corrosion, small sample size, virtual sample generation, Gaussian Mixture Model, generative adversarial network

## Abstract

Corrosion is the predominant failure mechanism in marine steel, and accurate corrosion prediction is essential for effective maintenance and protection strategies. However, the limited availability of corrosion datasets poses significant challenges to the accuracy and generalization of prediction models. This study introduces a novel integrated model designed for predicting marine corrosion under small sample sizes. The model utilizes dynamic marine environmental factors and material properties as inputs, with the corrosion rate as the output. Initially, a genetic algorithm (GA)-optimized machine learning framework is employed to derive the optimal GA-XGBoost model. To further enhance model performance, a virtual sample generation method combining Gaussian Mixture Model and Regression Generative Adversarial Network (GMM-RegGAN) is proposed. By incorporating these generated virtual samples into the base model, the prediction accuracy is further improved. The proposed framework is validated using corrosion datasets from six types of marine steel. Results demonstrate that GA optimization substantially improves both the performance and stability of the model. Virtual sample generation further enhances predictive performance, with reductions of 14.94% in RMSE, 15.55% in MAE, and 14.04% in MAPE. The results indicate that the proposed method offers a robust and effective framework for corrosion prediction in scenarios with limited sample data.

## 1. Introduction

Corrosion is one of the most significant challenges faced by metallic structures in marine engineering [1,2,3]. In marine environments, various metal structures, including ships, offshore platforms, and subsea pipelines, are particularly vulnerable to corrosion. Such failures can result in severe safety hazards and substantial economic losses [4,5,6]. Consequently, developing effective corrosion prediction methods is essential to enhance the safety and durability of marine structures.

Marine corrosion is a complex physical process influenced by the material elements and dynamic marine conditions [7,8,9]. As the demand for higher prediction accuracy increases, machine learning models have emerged as the primary approach for corrosion prediction [10,11]. However, the high time and economic costs associated with corrosion measurements often lead to datasets with small sample sizes [12,13,14]. With regard to the metal marine corrosion prediction with small sample sizes, current machine learning methods primarily encounter two key challenges: (1) the optimization of model hyperparameters and generalization ability; (2) the limitations on model prediction performance imposed by the sparsity of the small sample space.

In terms of model optimization, the hyperparameters of machine learning models largely determine their generalization ability and stability. In the domain of small-sample corrosion prediction, commonly used machine learning algorithms include partial least square (PLS) [15,16], support vector regression (SVR) [17,18,19], Gaussian process regression (GPR) [20,21], artificial neural networks (ANNs) [22,23,24], and ensemble learning models [8,21,25,26]. These algorithms perform well in corrosion prediction modeling due to their ability to capture complex relationships between input features and corrosion outcomes. However, their performance in small-sample problems is highly sensitive to hyperparameters. To enhance their performance, efficient hyperparameter optimization is crucial [27,28]. Traditional hyperparameter optimization methods, such as grid search and random search, are often inefficient in high-dimensional and small-sample settings. These approaches can become computationally expensive and are prone to being trapped in local optima, reducing their effectiveness [29,30]. In contrast, evolutionary algorithms, such as genetic algorithms, are well suited for tackling large-scale, high-dimensional optimization problems. The ability of evolutionary algorithms to search global optima and handle complex, non-linear optimization landscapes makes it an ideal candidate for improving model selection [31,32,33]. Although evolutionary algorithms have been gradually applied in regression prediction fields, there is considerable potential for expanding their use in small-sample marine corrosion prediction.

In terms of sample space sparsity, it is the primary constraint influencing further improvements in the accuracy of small-sample corrosion prediction. Virtual sample generation (VSG) technology, as an effective soft computing method, plays a key role in addressing the high-dimensional sample sparsity problem [34,35,36]. By utilizing a small number of real data, VSG technology generates new virtual samples based on statistical models or algorithms, thereby expanding the dataset and improving the performance of prediction models [37]. Currently, VSG techniques are primarily categorized into on noise injection-based, sample distribution-based, feature mapping-based, and generative adversarial network (GAN)-based methods. The noise introduction method typically generates new virtual sample inputs by adding random or white noise to the original samples [38,39]. The sample distribution-based method estimates the probability distribution of the original inputs and generates new virtual samples by randomly sampling from this estimated distribution [34,40,41,42]. The feature mapping-based method is analogous to sampling approaches. However, it first projects the original feature space into a specific feature space, followed by interpolation or sampling within this transformed space to generate virtual samples [43,44,45]. The GAN-based method trains a generator and a discriminator using the original dataset. Virtual samples are generated by sampling from the noise space and feeding them into the generator. Common variants of this approach include Conditional GAN (CGAN) and Regression GAN (RegGAN) [46,47,48,49,50]. Despite the advancements made by VSG in the domain of soft computing, its application in marine corrosion prediction remains considerably underdeveloped. Sutojo et al. [14] proposed a linear interpolation-based virtual sample generation method, which was validated in the assessment of corrosion inhibitor performance. Shen and Qian [51] utilized a Gaussian mixture model (GMM) for virtual sample generation and applied it to the degradation of rubber materials, resulting in a significant enhancement of aging modeling accuracy. These studies underscore the potential of VSG in addressing small-sample corrosion prediction challenges. However, further advancements are required in the validation of virtual sample effectiveness, particularly with regard to the multi-modal distribution characteristics inherent in marine corrosion processes.

Therefore, we propose an integrated machine learning framework for marine steel corrosion prediction that addresses two fundamental challenges inherent to small-sample scenarios: model generalization and predictive accuracy. The proposed framework combines GA-XGBoost and GMM-RegGAN. Initially, a genetic algorithm (GA) is employed to optimize the hyperparameters of the XGBoost model, thereby enhancing its generalization capability. Subsequently, a virtual sample generation method based on Gaussian Mixture Models and Regression Generative Adversarial Networks (GMM-RegGAN) is utilized to alleviate sample space sparsity and improve predictive performance. Through this integration, the proposed model aims to enable accurate and robust prediction of marine steel corrosion under limited data conditions. The proposed method was validated on the small-sample marine steel corrosion dataset collected in this study and achieved good modeling results.

## 2. Dataset Construction

### 2.1. Original Dataset Collection

In this study, marine corrosion data of six commonly used marine engineering structural steels were collected from the literature [52]. Each original data record includes environmental conditions of the experiment, material composition, and the corresponding corrosion rate. The six marine steels and their element information are shown in Table 1.

During the experiment, five environmental features were monitored and recorded: seawater temperature (*T*), seawater salinity (*Sal*), dissolved oxygen (*DO*), seawater pH, and oxidation–reduction potential (*ORP*). The variation ranges of each environmental factor in the experimental conditions were set to remain consistent with real marine environments. Seawater temperature was controlled using a thermostat, with a range of 10 to 35 °C. Salinity was adjusted by adding NaCl to distilled water, with a range of 5 to 45‰. Dissolved oxygen was varied by injecting different amounts of O_2_ and N_2_ into the seawater, with a range of 0 to 15 mg/L. Seawater pH was adjusted by adding HCl and NaOH, with a range of 5 to 10. ORP was controlled by adding hydrochloric acid and NaOH solution to the seawater, with a range of 100 to 450 mV. The corrosion rates of six marine steels in different environments were measured through electrochemical testing. Based on the Stern–Geary equation, the corrosion rate of the steel was calculated using the measured Tafel slopes and linear polarization resistance [53]. The detailed records of environmental factors and corrosion rates are listed in Supplementary Table S1.

### 2.2. Feature Engineering

Based on the feature creation methods proposed in the literature [54], we transformed the original metal element information into 17 types of physical, heat, atomic, electronegativity, and orbital properties. The descriptions and symbol definitions of these properties are shown in Table 2. For each element, the corresponding property values were obtained from [55]. The calculation formulas for feature creation are as follows:

(1) $$X_{-}=X_{B}/X_{C}=\sum_{i=1}^{n}f_{B_{i}}X^{B_{i}}/\sum_{i=1}^{m}f_{C_{i}}X^{C_{i}}$$

(2) $$X_{+}=X_{B}\cdot X_{C}=\left(\sum_{i=1}^{n}f_{B_{i}}X^{B_{i}}\right)\cdot \left(\sum_{i=1}^{m}f_{C_{i}}X^{C_{i}}\right)$$

where *X* represents one of the properties, *B* denotes non-metallic elements, *C* represents metallic elements, $f_{B_{i}}$ and $f_{C_{i}}$ are the mass fraction of the metallic element $B_{i}$ and non-metallic element $C_{i}$, $X^{B_{i}}$ and $X^{C_{i}}$ represent the property values of the corresponding metallic and non-metallic element, and *m* and *n* indicate the number of non-metallic and metallic elements.

Using the above feature creation method, each property is defined as 2 new features (i.e., $X_{-}$ and $X_{+}$), resulting in the generation of 34 new features.

### 2.3. Feature Reduction

Due to the high dimensionality after feature engineering, potential multi-collinearity issues may arise [56,57]. Therefore, we performed feature reduction analysis. Features were selected using correlation analysis, variance analysis, and feature importance analysis methods. The specific implementation is as follows:

**(1)** Correlation feature set creation based on Pearson correlation analysis. The Pearson correlation coefficient between each pair of features is calculated as follows:

(3) $$\rho \left(X_{i},X_{j}\right)=\frac{\sum_{k=1}^{n}\left(X_{i}^{\left(k\right)}-{\bar{X}}_{i}\right)\left(X_{j}^{\left(k\right)}-{\bar{X}}_{j}\right)}{\sqrt{\sum_{k=1}^{n}{\left(X_{i}^{\left(k\right)}-{\bar{X}}_{i}\right)}^{2}}\sqrt{\sum_{k=1}^{n}{\left(X_{j}^{\left(k\right)}-{\bar{X}}_{j}\right)}^{2}}}$$

where $\rho \left(X_{i},X_{j}\right)$ is the Pearson correlation coefficient, and ${\bar{X}}_{i}$ is the mean value of $X_{i}$.

Features with correlation coefficients greater than 0.9 are grouped into set $G=\left\{G_{1},G_{2},\cdots ,G_{K}\right\}$, where each subset $G_{k}$ satisfies the following condition:

(4) $$G_{k}=\left\{X_{i}|\rho \left(X_{i},X_{j}\right)\geq 0.9\;\mathrm{for}\;\mathrm{some}\;X_{j}\in G_{k}\right\}$$

**(2)** Feature selection based on variance analysis. For each subset $G_{k}=\left\{X_{i_{1}},X_{i_{2}},\cdots ,X_{i_{k}}\right\}$, the variance of each feature $Var\left(X_{i_{k}}\right)$ is calculated. Feature $X_{i^{*}}$ with the highest variance is then retained for each subset.

(5) $$X_{i^{*}}=\underset{X_{i_{k}}\in G_{k}}{\arg\max}Var\left(X_{i_{k}}\right)$$

**(3)** Feature reduction based on GBDT feature importance analysis. By evaluating the contribution of each feature to the splits in the decision tree, the importance of each feature is recorded as $w_{i}$, where $\sum_{i=1}^{p}w_{i}=1$. The features are then arranged in descending order and denoted as $w_{\left(i\right)}$. To select features with cumulative importance exceeding the threshold θ (set 0.9), the calculation formula is as follows:

(6) $$S=\left\{i|\sum_{i\in S}w_{\left(i\right)}\geq \theta \right\}$$

where S is the final feature set after reduction.

Following the dimensionality reduction process, five environmental features (*T*, *DO*, *Sal*, *pH*, *ORP*) and six property features (*ENs*^+^, Δ*H_f_*^+^, *T_m_*^+^, *V*^−^, Δ*H_v_*^+^, *R_m_*^−^) were retained, effectively mitigating issues associated with feature dimensionality and multi-collinearity. A comprehensive presentation of the results and analysis to the feature creation and reduction process can be found in Supplementary Text S1.

## 3. Integrated Machine Learning Method

**Figure 1:**
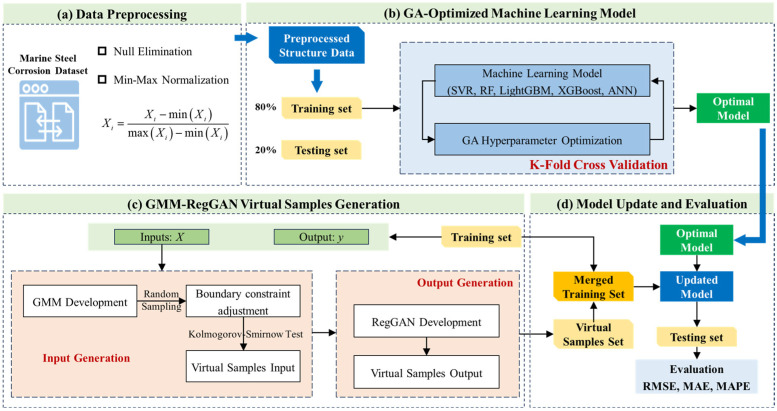
Holistic framework of the proposed machine learning method.

For the problem of small-sample marine steel corrosion prediction in dynamic environments, the holistic framework of the proposed machine learning method is shown in Figure 1, which consists of four parts: (a) Data preprocessing; (b) GA-optimized machine learning model; (c) GMM-RegGAN virtual samples generation; and (d) model update and evaluation. A detailed description of each part is provided below.

### 3.1. Data Preprocessing

This section presents the data preprocessing of the marine steel corrosion dataset. First, data with incomplete feature records were handled by deletion. For the corrosion dataset after feature engineering and reduction, there are significant differences in the value ranges of environmental and property features. These differences in feature ranges can lead to low model training efficiency and instability in numerical precision [56]. Therefore, we applied the Min-Max normalization method to process each feature, with the following calculation formula:

(7) $$X_{i}=\frac{X_{i}-\min\left(X_{i}\right)}{\max\left(X_{i}\right)-\min\left(X_{i}\right)}$$

where $X_{i}$ is the *i*-th feature, and $\min\left(\cdot \right)$ and $\max\left(\cdot \right)$ represent the minimum and maximum values of the corresponding feature, respectively.

### 3.2. GA-Optimized Machine Learning Model

**Figure 2:**
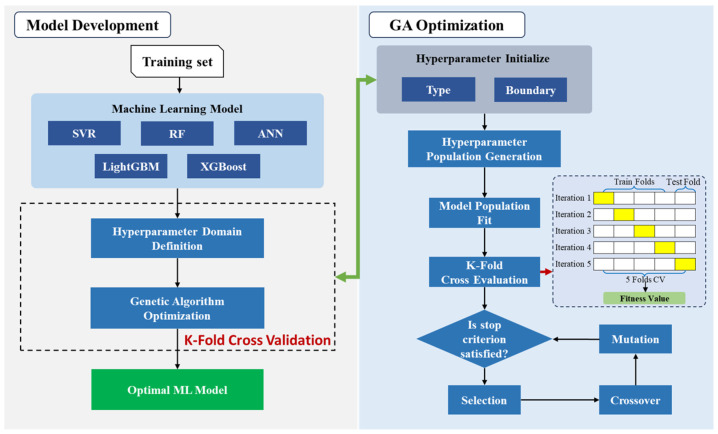
GA-optimized machine learning model development.

The preprocessed structure dataset is split into a training set (80%) and a testing set (20%). The training set is used for model development, while the testing set evaluates the model prediction and generalization performance. The specific process of GA-optimized machine learning model development is illustrated in Figure 2.

Five most commonly used machine learning algorithms, including SVR, RF, LightGBM, XGBoost, and ANN, are selected as candidate models. In this study, the genetic algorithm (GA), a global search evolutionary algorithm, was selected for hyperparameter tuning [31]. The selected five models have distinct hyperparameters, and the ranges of these hyperparameters were informed by relevant research [33,58,59]. Meanwhile, to maximize the potential for finding optimal hyperparameters, the ranges were expanded as much as possible. The hyperparameters of each model and their variation ranges are listed in Table 3.

Based on the training set, we used the *K*-fold cross-validation method to evaluate the model tuning results. The formula for selecting the hyperparameters of model is defined as follows:

(8) $$\theta =\underset{\theta \in \Omega }{\arg\max}\frac{1}{K}\sum_{k=1}^{K}L\left(y_{k},f\left(X_{k};\theta \right)\right)$$

where K is the number of folds in the cross-validation, θ is the hyperparameter vector, Ω is the hyperparameter space, $X_{k}$ is the input of the *k*-th fold training set sample, $y_{k}$ is the output of the *k*-th fold training set sample, $f\left(X_{k};\theta \right)$ is the predicted value, and $L\left(\cdot \right)$ is the loss function.

As can be seen from Figure 2, the GA starts by randomly selecting the initial sample population from the hyperparameter sample space and encoding it. Based on the fitness of each hyperparameter set, GA performs selection, crossover, and mutation operations to obtain new hyperparameter populations. The hyperparameter optimization process iterates continuously until the stopping criterion is met, and the optimal machine learning model is output. The stopping criterion set in this study for the hyperparameter search is that the optimal prediction error between two generations of hyperparameter populations should be less than 0.01, at which point the optimization stops. Finally, the optimal model is selected as the best model after GA tuning from all the preselected models.

### 3.3. GMM-RegGAN Virtual Sample Generation

**Figure 3:**
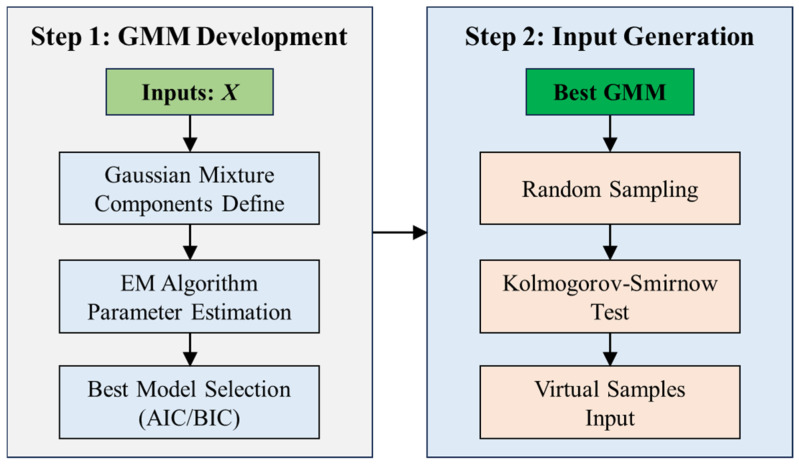
Method of virtual sample input generation.

As can be seen from Figure 3, the GMM-RegGAN virtual sample generation method proposed in this study is divided into two parts: (1) virtual sample input generation and (2) virtual sample output generation. The specific implementation methods are as follows.

#### 3.3.1. Input Generation Based on GMM

The generation of virtual sample inputs is primarily achieved through sampling from a Gaussian Mixture Model (GMM). The method for generating virtual sample inputs, as shown in Figure 3, involves two steps: GMM development and input generation.

**Step 1.** GMM development. For the input dataset $X=\left\{x_{1},x_{2},\cdots ,x_{N}\right\}$, where $x_{i}\in {\mathbb{R}}^{m}$, it is assumed to follow a mixture model of *K* Gaussian distributions. The probability density function (PDF) of GMM can be expressed as follows:

(9) $$p\left(x\right)=\sum_{k=1}^{K}\pi_{k}f\left(x|\mu_{k},\sum_{k}\right)$$

where $\pi_{k}$ represents the weight of the *k*-th Gaussian component, satisfying $\sum_{k=1}^{K}\pi_{k}=1$ and $\pi_{k}\geq 0$, $f\left(x|\mu_{k},\sum_{k}\right)$ is the PDF of the *k*-th Gaussian distribution, defined as follows:

(10) $$f\left(x|\mu_{k},\sum_{k}\right)=\frac{1}{{\left(2\pi \right)}^{d/2}{\left|\Sigma_{k}\right|}^{1/2}}\exp\left(-\frac{1}{2}{\left(x-\mu_{k}\right)}^{T}\Sigma_{k}^{-1}\left(x-\mu_{k}\right)\right)$$

where $\mu_{k}$ and $\Sigma_{k}$ are the mean vector and covariance matrix of the distribution. For the parameters $\left\{\pi_{k},\mu_{k},\Sigma_{k}\right\}$, the Expectation–Maximization (EM) algorithm is used to solve them. The iterative process of the E-step and M-step is as follows:

- E-step (Expectation Step): In this step, we compute the posterior probability that each data point belongs to the *k*-th Gaussian component. The posterior probability, also known as the responsibility $\gamma_{ik}$, is calculated as follows:
  (11) $$\gamma_{ik}=\frac{\pi_{k}f\left(x_{i}|\mu_{k},\sum_{k}\right)}{\sum_{j=1}^{K}\pi_{j}f\left(x_{i}|\mu_{j},\sum_{j}\right)}$$
- M-step (Maximization Step): In this step, the model parameters are updated to maximize the log-likelihood function. The parameters $\left\{\pi_{k},\mu_{k},\Sigma_{k}\right\}$ are updated as follows:
  (12) $$\pi_{k}=\frac{1}{N}\sum_{i=1}^{N}\gamma_{ik}$$
  (13) $$\mu_{k}=\sum_{i=1}^{N}\gamma_{ik}x_{i}/\sum_{i=1}^{N}\gamma_{ik}$$
  (14) $$\Sigma_{k}=\frac{\sum_{i=1}^{N}\gamma_{ik}\left(x_{i}-\mu_{k}\right){\left(x_{i}-\mu_{k}\right)}^{T}}{\sum_{i=1}^{N}\gamma_{ik}}$$

Finally, the selection of the best model is based on the Akaike Information Criterion (AIC) and the Bayesian Information Criterion (BIC) to evaluate the performance of models for different Gaussian component values of *K*.

**Step 2.** Input generation based on the best GMM. After obtaining the optimal GMM model, we generate the required virtual sample input by performing random sampling from the best model. To ensure the validity of the virtual samples, we first remove unreasonable samples through boundary constraints. The distribution range of all generated samples is constrained to stay within the range of the training samples. Then, to ensure that the generated virtual samples match the statistical characteristics of the original data, we use the Kolmogorov–Smirnov (K-S) test to evaluate the consistency between the generated sample distribution and the original distribution. The K-S test statistic is defined as follows:

(15) $$D=\underset{x}{\sup}\left|F_{\tilde{X}}(x)-F_{X}(x)\right|$$

where $F_{\tilde{X}}\left(x\right)$ and $F_{X}\left(x\right)$ are the empirical cumulative distribution functions (CDFs) of the generated samples and original samples, respectively. If *D* is smaller than the critical value, the generated virtual samples are considered valid.

#### 3.3.2. Output Generation Based on RegGAN

**Figure 4:**
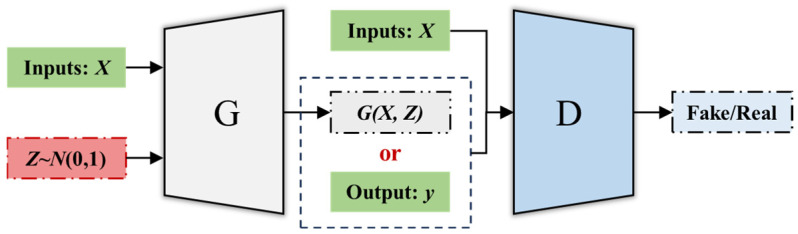
The model structure of RegGAN.

The output of the virtual samples is primarily obtained by feeding the virtual sample inputs into the RegGAN surrogate model. The structure of the RegGAN model constructed in this study is illustrated in Figure 4.

In Figure 4, both the generator (G) and discriminator (D) are three-layer neural networks. The specific hyperparameters are defined in Table 4. The input to the G consists of the true labels from the training set samples and random noise, and the output is the generated mapped sample. The input to D includes the true inputs of the training set samples, the outputs from the generator, and the labels of the real samples. The output of the discriminator is a judgment of whether the samples are real or generated (virtual).

During training, the generator is updated twice for every one update of the discriminator. The training losses for the generator and discriminator are defined as follows:

(16) $$L_{G}=-\frac{1}{N}\sum_{i=1}^{N}\log\left(D\left(x_{i},G\left(z_{i},x_{i}\right)\right)\right)$$

(17) $$L_{D}=-\frac{1}{2N}\sum_{i=1}^{N}\left[\log\left(D\left(x_{i},y_{i}\right)\right)+\log\left(1-D\left(x_{i},G\left(z_{i},x_{i}\right)\right)\right)\right]$$

where $D\left(x_{i},G\left(z_{i},x_{i}\right)\right)$ and $D\left(x_{i},y_{i}\right)$ represent the probabilities that the discriminator classifies real samples and generated (virtual) samples as real, respectively.

For the output of the virtual samples, we obtain $y_{vir}=G\left(z_{vir},x_{vir}\right)$ by inputting the generated input $x_{vir}$ from the GMM model and the randomly sampled noise $z_{vir}$ into the generator (G) of the trained RegGAN. To address the variability introduced by the random sampling noise, we perform 20 random samplings for each input and compute the mean of the resulting outputs. This mean is then taken as the final output for the virtual sample.

### 3.4. Model Update and Evaluation

After generating the virtual sample set, the training set samples and the generated virtual samples are merged to form a new training set. The optimal base model is then re-trained and the parameters are updated using the new dataset.

The performance of the proposed model is ultimately evaluated on the testing set. Three evaluation metrics are selected: mean absolute error (MAE), root mean squared error (RMSE), and mean percentage error (MPE). The formulas for these evaluation metrics are as follows:

(18) $$\mathrm{MAE}=\frac{1}{n}\sum_{i=1}^{n}\left|y_{i}-{\hat{y}}_{i}\right|$$

(19) $$\mathrm{RMSE}=\sqrt{\frac{1}{n}\sum_{i=1}^{n}{\left(y_{i}-{\hat{y}}_{i}\right)}^{2}}$$

(20) $$\mathrm{MAPE}=\frac{1}{n}\sum_{i=1}^{n}\left|\frac{y_{i}-{\hat{y}}_{i}}{y_{i}}\right|$$

where *n* is number of samples, $y_{i}$ is the true value of the *i*-th sample, and ${\hat{y}}_{i}$ is the predicted value of the *i*-th sample. To further assess the improvement in performance due to the VSG technique, the error improvement rate (EIR) metric is proposed. This metric quantifies the degree of performance improvement in the machine learning model. The calculation formula for the EIR is as follows:

(21) $$\mathrm{EIR}\text{\_}\mathrm{MAE}=\frac{{\mathrm{MAE}}_{before}-{\mathrm{MAE}}_{after}}{{\mathrm{MAE}}_{before}}$$

(22) $$\mathrm{EIR}\text{\_}\mathrm{RMSE}=\frac{{\mathrm{RMSE}}_{before}-{\mathrm{RMSE}}_{after}}{{\mathrm{RMSE}}_{before}}$$

(23) $$\mathrm{EIR}\text{\_}\mathrm{MAPE}=\frac{{\mathrm{MAPE}}_{before}-{\mathrm{MAPE}}_{after}}{{\mathrm{MAPE}}_{before}}$$

where ${\mathrm{MAE}}_{before}$, ${\mathrm{RMSE}}_{before}$, and ${\mathrm{MAPE}}_{before}$ refers to the evaluation result when the model is built using only the original training set samples, and ${\mathrm{MAE}}_{after}$, ${\mathrm{RMSE}}_{after}$, and ${\mathrm{MAPE}}_{after}$ refers to the evaluation result after updating the model with virtual samples.

Considering the inherent randomness in virtual sample generation, all virtual sample generation methods in this study were repeated 50 times, and the average value was taken as the evaluation result. This approach helps mitigate the variability introduced by random sampling and provides a more stable and reliable performance evaluation.

## 4. Results

### 4.1. GA-Optimized Machine Learning Model Development and Validation

**Figure 5:**
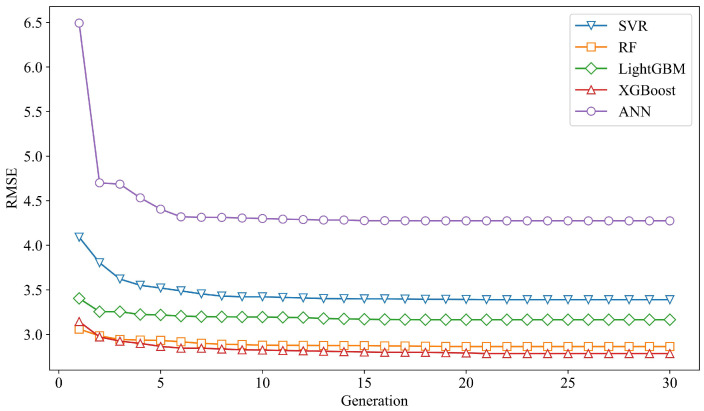
Model performance changes in the GA optimization process.

The marine steel corrosion dataset was divided into a training set and a test set, with 80% allocated for training and 20% for testing. Following the GA-based machine learning optimization framework proposed in Section 3.2, we developed and fine-tuned five classical machine learning algorithms: SVR, RF, LightGBM, XGBoost, and ANN. During the hyperparameter optimization process, 5-fold cross-validation was employed to evaluate model performance, with the root mean square error (RMSE) used as the optimization objective. The performance variations of the five algorithms during the GA optimization process are illustrated in Figure 5.

**Figure 6:**
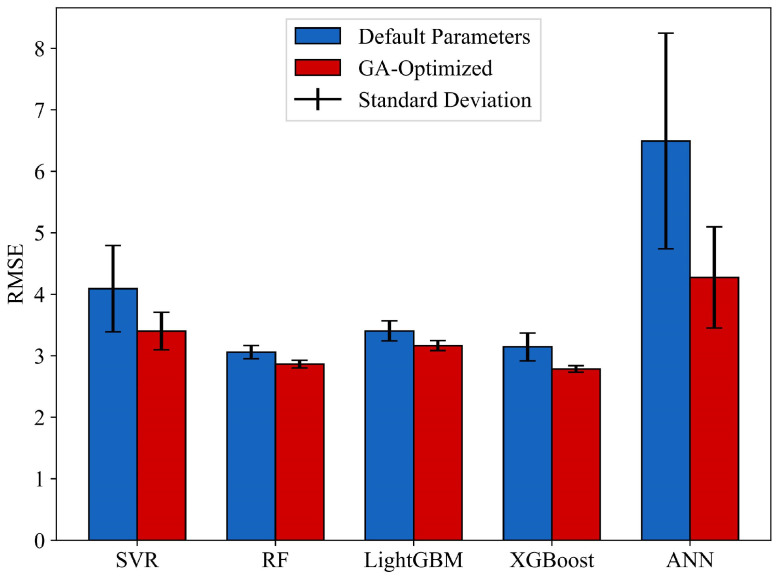
Cross-validation performance results of GA hyperparameter optimization.

As shown in the figure, the RMSE values of all five models decrease rapidly during the initial generations of the GA iterations, followed by a gradual deceleration in the rate of decline. With continued iterations, the RMSE values of the five models progressively stabilize and eventually converge. Table S2 lists the final hyperparameters of the five models after GA-based optimization. In addition, we compared the mean and variance of the cross-validated RMSE on the training set before and after GA optimization for each model. The corresponding results are presented in Figure 6 and Table 5.

As can be seen from the figure, after GA optimization, the performance of all five models significantly improved, and their variance decreased substantially. The results suggest that GA-based hyperparameter optimization significantly enhanced both model performance and stability. Meanwhile, RF, LightGBM, and XGBoost outperformed SVR and ANN, highlighting the advantages of ensemble learning models in small-sample problems. Under default hyperparameters, the RF model achieved the best performance, with a mean RMSE of 3.060 and a standard deviation of 0.107. Among the optimized models, the GA-XGBoost model demonstrated the best performance, with a cross-validation RMSE of 2.785 and a standard deviation of 0.054. Based on the results, GA-XGBoost was selected as the optimal model for developing the marine steel corrosion prediction model.

### 4.2. GMM Development and VSG Input Analysis

**Figure 7:**
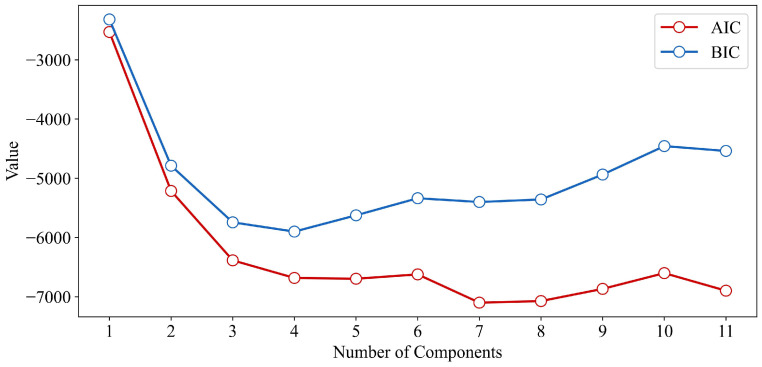
AIC and BIC values for GMMs with different numbers of components.

For the input features of the training set, we first developed a Gaussian Mixture Model (GMM) to generate virtual sample input and evaluate their rationality. Considering that the input feature dimension of the dataset is 11, the number of GMM components was set from 1 to 11. For each GMM configuration, parameter estimation was performed using the EM algorithm, and model fit was quantified using the AIC and the BIC metrics. The evaluation metrics for GMMs with different numbers of components are shown in Figure 7.

As shown in the figure, both AIC and BIC values exhibit a trend of initially decreasing and then increasing as the number of components increases. In the early stages, the sharp decline in AIC/BIC suggests that the GMM was initially unable to fully capture the multi-modal characteristics of the input features. However, as the number of components continues to grow, the AIC and BIC values begin to rise, indicating an increased risk of overfitting due to excessive model complexity, which is detrimental to generalization. According to the AIC results, the improvements become marginal after four components, with the lowest AIC value observed at seven components. In contrast, the BIC reaches its minimum at four components and subsequently increases as model complexity rises. Considering both model complexity and performance, we ultimately selected the GMM with four components as the optimal model.

**Figure 8:**
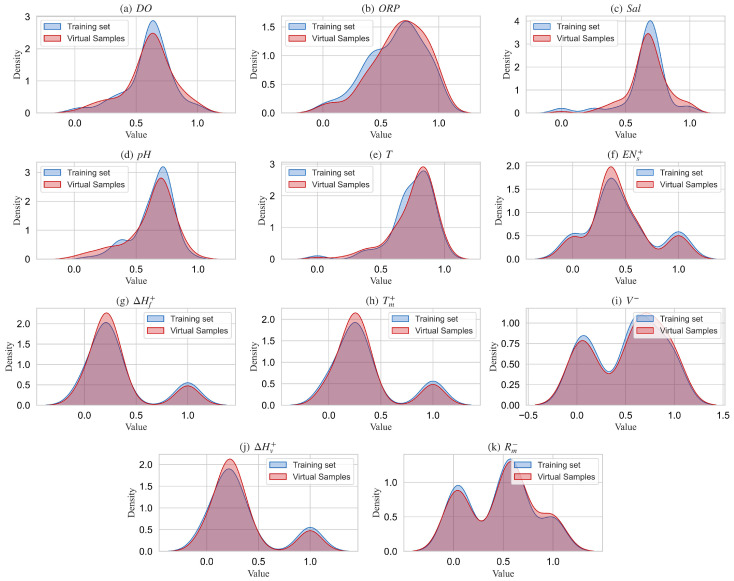
Comparison of feature distributions between virtual samples and real training samples.

After the development of the GMM, the virtual sample input was generated by randomly sampling from the optimal GMM model. The boundaries of the generated virtual samples were constrained within the feature boundaries of the training set. To validate whether the distribution of the generated virtual samples is consistent with that of the real training samples, we generated a set of virtual samples (100 samples) approximately matching the size of the training set. Figure 8 shows the probability density distributions for each feature in both the generated virtual samples and the real training samples.

As can be seen from the figure, the distribution of the virtual samples sampled from the best GMM model shows a good correspondence with the real training sample distribution, particularly in recognizing the multi-modal characteristics of the features. The GMM successfully captured the multi-modal nature of the 11 features, with the peak characteristics of the virtual samples maintaining a high degree of consistency with those of the real training samples. Furthermore, we performed a K-S test to quantitatively assess the consistency between the distributions of the virtual and real samples, and the results are presented in Table 6.

As can be seen from the table, the mean and variance of the virtual samples are quite close to those of the real samples. In the K-S test, the D-values for all features are below 0.22. Using a significance level of 0.05 as the test criterion, all the 11 features passed the distribution consistency test. The results further quantitatively validate that the GMM model accurately captures the multi-modal distribution characteristics of the original training set samples and can be used to generate realistic virtual sample inputs.

### 4.3. RegGAN Training and VSG Output Analysis

**Figure 9:**
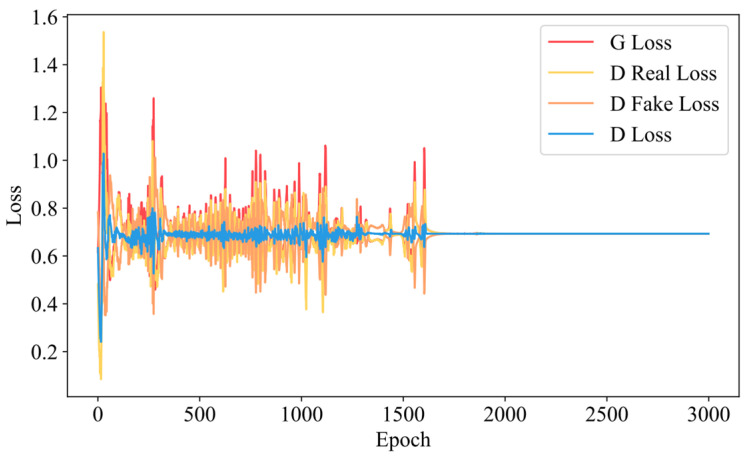
Training loss variation of the RegGAN.

Based on the RegGAN structure defined in Section 3.3.2, we performed model parameter estimation and evaluation using the training set data. During training, the generator is updated twice for every one update of the discriminator. Figure 9 illustrates the changes in the losses of the generator and discriminator during the training process of the RegGAN model.

In the figure, the red line represents the generator loss, the yellow and orange lines represent the discriminator losses for real and virtual samples, respectively, and the blue line indicates the average discriminator loss. As can be seen from the figure, in the early stages of training, both the generator and discriminator losses are relatively high. As training progresses, both the generator and discriminator losses decrease, accompanied by significant fluctuations. In the final stages of training, the losses of both the generator and discriminator converge close to log(0.5), indicating that the losses of both the generator and discriminator have stabilized.

**Figure 10:**
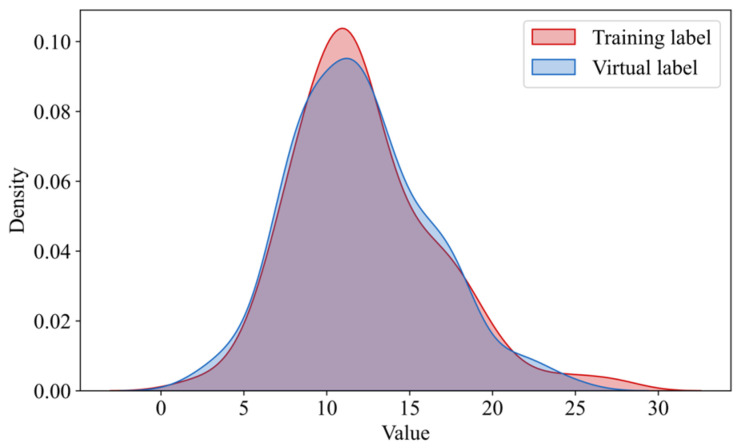
Comparison of the output distribution between virtual samples and real training samples.

Using the virtual samples generated by the GMM model in Section 4.2 as input, and combining them with random sampling from the noise space, we feed them into the generator of RegGAN to produce virtual sample outputs. To address the variability introduced by random sampling noise, we perform 20 random samplings for each input and compute the mean of the resulting outputs. Figure 10 shows the probability density functions of the virtual sample outputs and the training sample outputs. As observed in the figure, the generated virtual sample outputs exhibit a high degree of consistency with the training set samples, validating the reasonableness of the generated virtual samples.

### 4.4. Model Update and Performance Comparison

**Figure 11:**
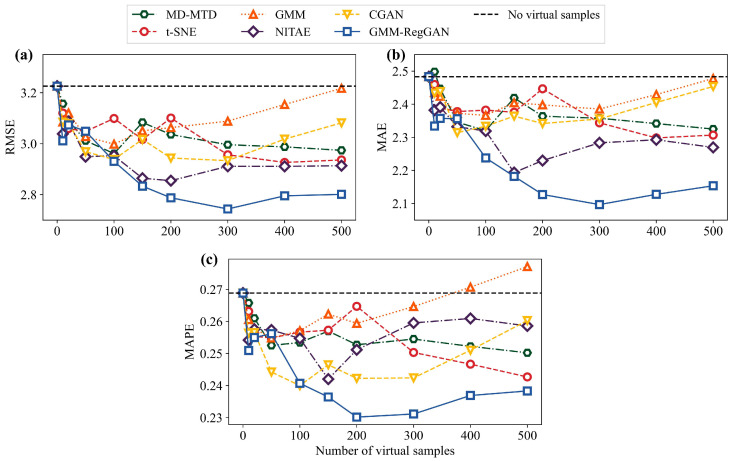
Prediction performance of the proposed model and the comparative models under varying quantities of virtual samples. (**a**) RMSE, (**b**) MAE, and (**c**) MAPE.

By setting the number of virtual samples to 0, 10, 20, 50, 100, 150, 200, 300, 400, and 500, we initially investigated the impact of varying quantities of virtual samples on the improvement of model performance. To better compare the optimization effects of the proposed virtual sample generation method, we selected five classical virtual sample generation models for comparison: MD-MTD [34], t-SNE [44], GMM [60], NITAE [45], and CGAN [46]. The performance improvements of the proposed model and the comparative models on the training set under different virtual sample quantities are illustrated in Figure 11 and Tables S3–S5.

As can be seen from the figure, the introduction of virtual samples for model updating generally leads to a reduction in the three-evaluation metrics across all the VSG methods. This demonstrates the feasibility of VSG methods in enhancing the prediction performance of corrosion in marine steel under small-sample conditions. Furthermore, different VSG methods result in varying optimal numbers of virtual samples for improving model performance. For most models, the optimal performance is achieved with between 100 and 300 virtual samples. Compared to other methods such as MD-MTD, t-SNE, GMM, NITAE, and CGAN, the proposed GMM-RegGAN model demonstrates the most significant improvement in performance, with notable reductions in the three metrics.

### 4.5. Performance Improvement Rate Analysis

**Figure 12:**
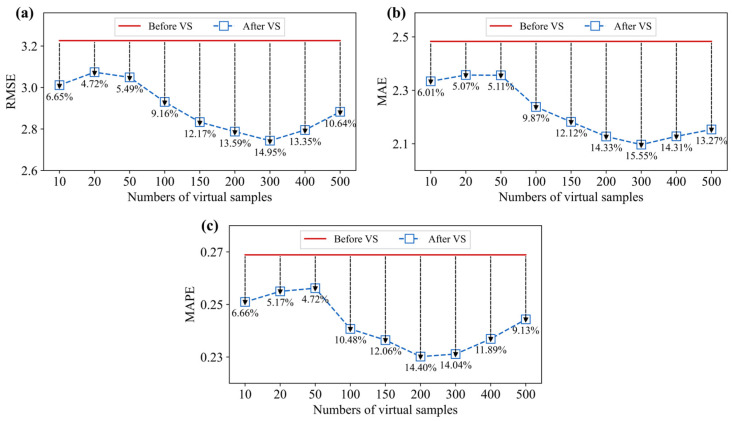
EIR improvement of different number of virtual samples. (**a**) RMSE, (**b**) MAE, and (**c**) MAPE.

To further quantitatively analyze the model performance improvement, we calculated the error improvement rate (EIR) of the evaluation metrics for different numbers of virtual samples. The results are shown in Figure 12.

From the figure, it can be seen that the performance of the proposed method followed a trend where the performance initially increases and then decreases as the number of virtual samples increases. The optimal performance was reached when the number of virtual samples is 300. When comparing the model with the original base model (without virtual sample) at the optimal number of virtual samples, the RMSE decreases from 3.226 to 2.744, MAE decreases from 2.483 to 2.097, and MAPE decreases from 0.269 to 0.231. The model EIRs for the three metrics are 14.95%, 15.55%, and 14.04%, respectively, indicating that the proposed model has good optimization performance.

**Figure 13:**
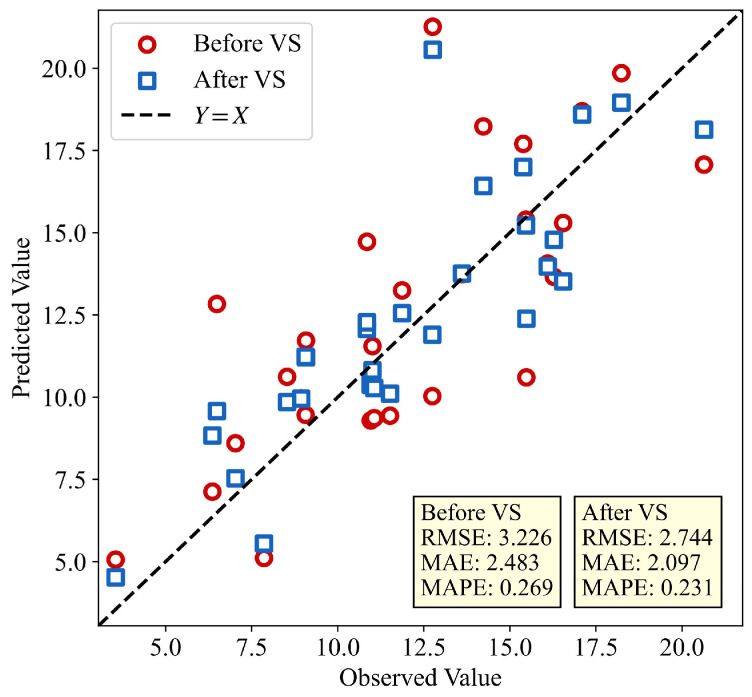
Prediction results of testing set samples before and after VSG.

To observe the improvement in single-sample prediction results after VSG, we used the GMM-RegGAN model to generate the optimal number of virtual samples. Then, we compared the prediction results for single testing samples with those of the base model. The results are shown in Figure 13. As can be seen from the figure, the model after VSG produces prediction results that were mostly between the predictions of base models and the actual values. This indicates that the virtual samples achieved improvement in the prediction error for individual samples.

## 5. Discussion

### 5.1. Approaches to Model Improvement

Virtual sample generation (VSG) is an effective approach to enhance model performance in small-sample problems using machine learning. However, there is a theoretical upper limit to the performance improvement provided by VSG, and the enhancement is not infinite. On one hand, the quality of the generated virtual samples directly impacts the model’s performance. If the virtual samples significantly deviate from the true data distribution or introduce noise, it may lead to overfitting and reduce the model’s generalization ability. On the other hand, VSG cannot generate entirely new information, it can only interpolate or extrapolate based on the existing data distribution. Thus, it cannot overcome the inherent limitations of the data itself. To further improve model performance, VSG can be combined with methods such as multi-model fusion and transfer learning. For example, by using transfer learning, degradation data from other domains can be transferred to the target task, optimizing feature extraction methods and enhancing the model’s generalization ability. The multi-model fusion strategy can also help avoid the limitations of single-model predictions. Combining these fusion modeling techniques provides an effective way to improve the prediction performance of small-sample marine steel corrosion.

### 5.2. Insight of the Model Structure

This study aims to address the challenges in small-sample marine steel corrosion prediction, including high-dimensional collinearity, poor model robustness, and low accuracy. The genetic algorithm-optimized machine learning framework overcomes model robustness issues in small-sample problems, significantly improving performance and stability. Meanwhile, the GMM-RegGAN virtual sample generation method updates the model by generating virtual samples, further enhancing its predictive accuracy. The proposed model structure has been validated on marine steel corrosion datasets, demonstrating its effectiveness. Given the difficulty in monitoring structural degradation in marine environments, most degradation issues in marine engineering share small-sample characteristics. Therefore, the proposed method is also applicable to various marine engineering problems with similar structures. Moreover, the current model proposed in this study only considers metal properties and dynamic marine environments as inputs. In reality, corrosion degradation of metals under various operating conditions and long-term service environments may also face small-sample modeling issues. Future research could expand the applicability of the proposed model by incorporating an analysis of factors such as operating conditions and service time.

## 6. Conclusions

This study proposes a novel integrated machine learning approach for predicting the corrosion of marine steel, aiming to enhance prediction accuracy with small sample sizes. The main contributions of this paper are as follows:

(1) To address the complex non-linear relationships among marine corrosion, environmental features, and metal properties, a genetic algorithm-optimized XGBoost (GA-XGBoost) model is proposed.
(2) To handle the small sample size characteristic of corrosion monitoring data, a virtual sample generation method combining a Gaussian Mixture Model and Regression Generative Adversarial Neural Network (GMM-RegGAN) is introduced.
(3) The proposed integrated model is validated on a collected marine steel corrosion dataset. GA effectively improves the baseline predictive performance, with the cross-validation RMSE decreasing by 12.58%. After augmenting the dataset with virtual samples and updating the model, RMSE, MAE, and MAPE were further reduced by 14.94%, 15.55%, and 16.96%, respectively.
(4) The integrated model is a general framework designed for small-sample corrosion prediction. It can be applied to the corrosion resistance evaluation of various types of metals and to classify the corrosion severity levels of the dynamic marine environment.

For marine corrosion prediction, based on the use of virtual sample generation techniques to address sample sparsity, future research can further focus on enhancing model interpretability and uncertainty quantification.

## Figures and Tables

**Table 1:** Compositional elements of the 6 typical marine steels.

Steel	C	Si	Mn	P	S	Cr	Mo	Ni	Cu	V	Fe
3C	0.180	0.300	0.300	0.400	0.400	0.300	0.002	0.300	0.035	0.010	Bal.
A3	0.160	0.300	0.500	0.045	0.050	0.025	0.001	0.013	0.031	0.001	Bal.
16Mn	0.300	0.200	1.400	0.035	0.030	0.000	0.000	0.015	0.000	0.020	Bal.
10MnPNbRe	0.140	0.200	0.900	0.080	0.045	0.300	0.001	0.300	0.300	0.000	Bal.
10CrMoAl	0.350	0.200	0.400	0.200	0.250	0.500	0.450	0.136	0.004	0.700	Bal.
D36	0.400	0.170	0.500	0.035	0.035	0.250	0.001	0.250	0.250	0.020	Bal.

**Table 2:** List of properties used in the feature engineering.

Feature Type	Symbol	Feature Description
Physical Property	*ρ*	Density of solid (kg/m^3^)
	*V*	Molar volume (cm^3^)
	*K*	Bulk modulus (GPa)
	*λ*	Thermal conductivity (W/m/K)
Heat Property	*T_m_*	Melting point (K)
	Δ*H_f_*	Enthalpy of fusion (kJ/mol)
	Δ*H_v_*	Enthalpy of vaporization (kJ/mol)
	Δ*H_a_*	Enthalpy of atomization (kJ/mol)
Atomic Property	*R_a_*	Atomic radius (pm)
	*R_m_*	Molecular single bond covalent radius (pm)
	*R_c_*	Covalent radius (pm)
	*R_v_*	van der Waals radius (pm)
Electronegativity Property	*EN_p_*	Pauling electronegativity
	*EN_s_*	Sanderson electronegativity
	*EN_a_*	Allred Rochow electronegativity
Orbital Oroperty	*E*_1_	First ionization energy (kJ/mol)
	*E*_2_	Second ionization energy (kJ/mol)

**Table 3:** Hyperparameters of base models and the variation ranges.

Model	Hyperparameter	Domain Definition
SVR	C	[0.1, 10]
	gamma	[0.1, 1]
	kernel function	[linear, rbf, poly, sigmoid]
RF	n_estimators	[20, 200]
	max_depth	[2, 11]
	min_samples_split	[2, 5]
LightGBM	n_estimators	[20, 200]
	max_depth	[2, 11]
	learning_rate	[0.01, 1]
XGBoost	n_estimators	[20, 200]
	max_depth	[2, 11]
	learning_rate	[0.01, 1]
ANN	hidden_layer	[1, 3]
	nodes	[10, 100]
	activation	[logistic, tanh, relu]
	alpha	[0.001, 0.1]

**Table 4:** Hyperparameters of RegGAN.

Network	Hyperparameter	Value
Generator (G)	input_dim	11
	noise_dim	16
	hidden_layer_1_dim	32
	hidden_layer_2_dim	32
	output_dim	1
Discriminator (D)	input_dim	12
	hidden_layer_1_dim	32
	hidden_layer_2_dim	16
	output_dim	1
Both	epochs	3000
	learning_rate	0.001
	optimizer	Adam

**Table 5:** Cross-validation RMSE results of GA hyperparameter optimization.

Model	Default Parameter	GA-Optimized
SVR	4.091 ± 0.703	3.401 ± 0.304
RF	**3.060 ± 0.107**	2.864 ± 0.063
LightGBM	3.404 ± 0.162	3.165 ± 0.081
XGBoost	3.145 ± 0.226	**2.785 ± 0.054**
ANN	6.493 ± 1.754	4.274 ± 0.823

**Table 6:** K-S test results for feature distributions of real and virtual samples.

Feature	Real Samples		Virtual Samples		K-S Test	
	Mean	Std	Mean	Std	*D*	*p*
*DO*	0.605	0.036	0.611	0.041	0.091	0.721
*ORP*	0.627	0.058	0.618	0.050	0.104	0.559
*Sal*	0.656	0.031	0.676	0.029	0.162	0.104
*pH*	0.608	0.041	0.626	0.037	0.136	0.248
*T*	0.753	0.029	0.751	0.023	0.116	0.426
$EN_{s}^{+}$	0.452	0.092	0.475	0.085	0.157	0.123
$\Delta H_{f}^{+}$	0.328	0.103	0.345	0.102	0.154	0.138
$T_{m}^{+}$	0.353	0.100	0.372	0.097	0.154	0.138
$V^{-}$	0.516	0.123	0.536	0.121	0.152	0.145
$\Delta H_{v}^{+}$	0.339	0.103	0.357	0.101	0.154	0.138
$R_{m}^{-}$	0.462	0.113	0.481	0.112	0.150	0.157

## Data Availability

The original contributions presented in this study are included in the article/Supplementary Material. Further inquiries can be directed to the corresponding authors.

## Supplementary Materials

The following supporting information can be downloaded at: https://www.mdpi.com/article/10.3390/ma18163760/s1. Text S1: Feature engineering and reduction analysis process and results. Figure S1: Results of Pearson correlation analysis. Figure S2: Result of feature importance and cumulative importance after correlation-based dimensionality reduction. Table S1: Experiment dataset for the six marine steels. Table S2: Hyperparameters of base models and the variation ranges. Table S3: Model RMSE for different numbers of virtual samples. Table S4: Model MAE for different numbers of virtual samples. Table S5: Model MAPE for different numbers of virtual samples.

## Abbreviations

The following abbreviations are used in this manuscript:

GA	Genetic algorithm
GMM	Gaussian mixture model
GAN	Generative adversarial network
RegGAN	Regression generative adversarial network

## References

[1] Odeyemi O.O., Alaba P.A. (2024). Efficient and Reliable Corrosion Control for Subsea Assets: Challenges in the Design and Testing of Corrosion Probes in Aggressive Marine Environments. Corros. Rev..

[2] Orlikowski J., Szociński M., Żakowski K., Igliński P., Domańska K., Darowicki K. (2022). Actual Field Corrosion Rate of Offshore Structures in the Baltic Sea along Depth Profile from Water Surface to Sea Bed. Ocean Eng..

[3] Li X., Guo M., Zhang R., Chen G. (2022). A Data-Driven Prediction Model for Maximum Pitting Corrosion Depth of Subsea Oil Pipelines Using SSA-LSTM Approach. Ocean Eng..

[4] Hou B., Li X., Ma X., Du C., Zhang D., Zheng M., Xu W., Lu D., Ma F. (2017). The Cost of Corrosion in China. npj Mater. Degrad..

[5] Li X., Zhang D., Liu Z., Li Z., Du C., Dong C. (2015). Materials Science: Share Corrosion Data. Nature.

[6] Yang Y., Khan F., Thodi P., Abbassi R. (2017). Corrosion Induced Failure Analysis of Subsea Pipelines. Reliab. Eng. Syst. Saf..

[7] Chen Q., Ma X., Liu Y., Shangguan Y., Wang H., Cai Y., She Z. (2025). Estimation of Atmospheric Chloride Deposition and Its Corrosion Effect in the Coastal Region of China. Corros. Rev..

[8] Yang J., Zou D., Zhang M., Que Z., Liu T., Zhou A., Li Y. (2024). Marine Steel Corrosion Prediction and Zonation Using Feature Extraction and Machine Learning in the Seas around China. Ocean Eng..

[9] Wang R.-Y., Dou Z.-F., Liu Z.-H., Li N., Liu X.-R., Zhang W.-F. (2025). Research on Ultraviolet Degradation Behavior and Aging Mechanisms of Fluorosilicone Rubber in Simulated Tropical Marine Atmospheric Environment. Polym. Degrad. Stab..

[10] Coelho L.B., Zhang D., Van Ingelgem Y., Steckelmacher D., Nowé A., Terryn H. (2022). Reviewing Machine Learning of Corrosion Prediction in a Data-Oriented Perspective. npj Mater. Degrad..

[11] Imran M.M.H., Jamaludin S., Mohamad Ayob A.F. (2024). A Critical Review of Machine Learning Algorithms in Maritime, Offshore, and Oil & Gas Corrosion Research: A Comprehensive Analysis of ANN and RF Models. Ocean Eng..

[12] Herowati W., Prabowo W.A.E., Akrom M., Setiyanto N.A., Kurniawan A.W., Hidayat N.N., Sutojo T., Rustad S. (2024). Machine Learning for Pyrimidine Corrosion Inhibitor Small Dataset. Theor. Chem. Acc..

[13] Ji Y., Li N., Cheng Z., Fu X., Ao M., Li M., Sun X., Chowwanonthapunya T., Zhang D., Xiao K. (2022). Random Forest Incorporating Ab-Initio Calculations for Corrosion Rate Prediction with Small Sample al Alloys Data. npj Mater. Degrad..

[14] Sutojo T., Rustad S., Akrom M., Syukur A., Shidik G.F., Dipojono H.K. (2023). A Machine Learning Approach for Corrosion Small Datasets. npj Mater. Degrad..

[15] Alamri A.H., Alhazmi N. (2022). Development of Data Driven Machine Learning Models for the Prediction and Design of Pyrimidine Corrosion Inhibitors. J. Saudi Chem. Soc..

[16] Davoodi F., Ashrafizadeh F., Atapour M., Rikhtehgaran R. (2022). A Novel Approach for Evaluation of Load Bearing Capacity of Duplex Coatings on Aluminum Alloy Using PLS and SVR Models. Trans. Nonferrous Met. Soc. China.

[17] Lu Z., Si S., He K., Ren Y., Li S., Zhang S., Fu Y., Jia Q., Jiang H.B., Song H. (2022). Prediction of Mg Alloy Corrosion Based on Machine Learning Models. Adv. Mater. Sci. Eng..

[18] Moses A., Chen D., Wan P., Wang S. (2023). Prediction of Electrochemical Corrosion Behavior of Magnesium Alloy Using Machine Learning Methods. Mater. Today Commun..

[19] Pei S., Dai C., Yang X., Zhang L., Wang H., Zhang S., Han Y., Li Q., Wang J. (2024). Quantitative Prediction of Mg-RE-Ni Alloy Corrosion Behavior by Machine Learning. Corros. Sci..

[20] Liu Y., Song Y., Keller J., Bond P., Jiang G. (2017). Prediction of Concrete Corrosion in Sewers with Hybrid Gaussian Processes Regression Model. RSC Adv..

[21] Xiong X., Zhang N., Yang J., Chen T., Niu T. (2024). Machine Learning-Assisted Prediction of Corrosion Behavior of 7XXX Aluminum Alloys. Metals.

[22] Alcalá F.J., Custodio E. (2008). Atmospheric Chloride Deposition in Continental Spain. Hydrol. Processes.

[23] Rocabruno-Valdés C.I., González-Rodriguez J.G., Díaz-Blanco Y., Juantorena A.U., Muñoz-Ledo J.A., El-Hamzaoui Y., Hernández J.A. (2019). Corrosion Rate Prediction for Metals in Biodiesel Using Artificial Neural Networks. Renew. Energy.

[24] Wei X., Fu D., Chen M., Wu W., Wu D., Liu C. (2021). Data Mining to Effect of Key Alloying Elements on Corrosion Resistance of Low Alloy Steels in Sanya Seawater environmentAlloying Elements. J. Mater. Sci. Technol..

[25] Chen Q., Wang H., Ji H., Ma X., Cai Y. (2024). Data-Driven Atmospheric Corrosion Prediction Model for Alloys Based on a Two-Stage Machine Learning Approach. Process Saf. Environ. Prot..

[26] Wang J., Zhang Z., Liu X., Shao Y., Liu X., Wang H. (2024). Prediction and Interpretation of Concrete Corrosion Induced by Carbon Dioxide Using Machine Learning. Corros. Sci..

[27] Wang Y., Su F., Guo Y., Yang H., Ye Z., Wang L. (2022). Predicting the Microbiologically Induced Concrete Corrosion in Sewer Based on XGBoost Algorithm. Case Stud. Constr. Mater..

[28] Yang L., Shami A. (2020). On Hyperparameter Optimization of Machine Learning Algorithms: Theory and Practice. Neurocomputing.

[29] Song Y., Wang Q., Zhang X., Dong L., Bai S., Zeng D., Zhang Z., Zhang H., Xi Y. (2023). Interpretable Machine Learning for Maximum Corrosion Depth and Influence Factor Analysis. npj Mater. Degrad..

[30] Wang N., Song L., Fang H., Li B., Wang F. (2023). Multi-Parameter Maximum Corrosion Depth Prediction Model for Buried Pipelines Based on GSCV-XGBoost. IEEE Access.

[31] Chen Q., Wang H., Liu Y., Shangguan Y., Ma X., Cai Y. (2024). Interpretable Data-Driven Prediction Methods for Atmospheric Chloride Deposition Rate. Atmos. Environ..

[32] Mirjalili S. (2019). Evolutionary Algorithms and Neural Networks. Stud. Comput. Intell..

[33] Xie M., Zhao J., Pei X. (2023). Maintenance Strategy Optimization of Pipeline System with Multi-Stage Corrosion Defects Based on Heuristically Genetic Algorithm. Process Saf. Environ. Prot..

[34] Li D.-C., Wu C.-S., Tsai T.-I., Lina Y.-S. (2007). Using Mega-Trend-Diffusion and Artificial Samples in Small Data Set Learning for Early Flexible Manufacturing System Scheduling Knowledge. Comput. Oper. Res..

[35] Zang D., Liu J., Qu F. (2021). Pipeline Small Leak Detection Based on Virtual Sample Generation and Unified Feature Extraction. Measurement.

[36] Yu H., Fan X., Wang G., Xie Y. (2024). VSG3A2: A Genetic Algorithm-Based Virtual Sample Generation Approach Using Information Gain and Acceptance-Rejection Sampling. IEEE Trans. Evol. Comput..

[37] Maqbool A., Khalad A., Khan N.Z. (2024). Prediction of Corrosion Rate for Friction Stir Processed WE43 Alloy by Combining PSO-Based Virtual Sample Generation and Machine Learning. J. Magnesium Alloys.

[38] Suh J.S., Suh B.-C., Lee S.E., Bae J.H., Moon B.G. (2022). Quantitative Analysis of Mechanical Properties Associated with Aging Treatment and Microstructure in Mg-al-Zn Alloys through Machine Learning. J. Mater. Sci. Technol..

[39] Yang J., Yu X., Xie Z.-Q., Zhang J.-P. (2011). A Novel Virtual Sample Generation Method Based on Gaussian Distribution. Knowl.-Based Syst..

[40] Li D.-C., Chen C.-C., Chang C.-J., Lin W.-K. (2012). A Tree-Based-Trend-Diffusion Prediction Procedure for Small Sample Sets in the Early Stages of Manufacturing Systems. Expert Syst. Appl..

[41] Zhu Q.-X., Gong H.-F., Yuan X., Yan-Lin H. A Bootstrap Based Virtual Sample Generation Method for Improving the Accuracy of Modeling Complex Chemical Processes Using Small Datasets. Proceedings of the 2017 6th Data Driven Control and Learning Systems (DDCLS).

[42] El Bilali A., Lamane H., Taleb A., Nafii A. (2022). A Framework Based on Multivariate Distribution-Based Virtual Sample Generation and DNN for Predicting Water Quality with Small Data. J. Clean. Prod..

[43] Zhang X.H., Xu Y., He Y.L., Zhu Q.X. (2021). Novel Manifold Learning Based Virtual Sample Generation for Optimizing Soft Sensor with Small Data. ISA Trans..

[44] He Y.L., Hua Q., Zhu Q.X., Lu S. (2022). Enhanced Virtual Sample Generation Based on Manifold Features: Applications to Developing Soft Sensor Using Small Data. ISA Trans..

[45] Tian Y., Xu Y., Zhu Q.-X., He Y.-L. (2021). Novel Virtual Sample Generation Using Target-Relevant Autoencoder for Small Data-Based Soft Sensor. IEEE Trans. Instrum. Meas..

[46] Zhu Q.-X., Hou K.-R., Chen Z.-S., Gao Z.-S., Xu Y., He Y.-L. (2021). Novel Virtual Sample Generation Using Conditional GAN for Developing Soft Sensor with Small Data. Eng. Appl. Artif. Intell..

[47] Iyer R.S., Iyer N.S., P R.A., Joseph A. (2024). Harnessing Machine Learning and Virtual Sample Generation for Corrosion Studies of 2-Alkyl Benzimidazole Scaffold Small Dataset with an Experimental Validation. J. Mol. Struct..

[48] Chen Z.-S., Hou K.-R., Zhu M.-Y., Xu Y., Zhu Q.-X. (2021). A Virtual Sample Generation Approach Based on a Modified Conditional GAN and Centroidal Voronoi Tessellation Sampling to Cope with Small Sample Size Problems: Application to Soft Sensing for Chemical Process. Appl. Soft Comput..

[49] Wang Y., Yan P. (2024). RegGAN: A Virtual Sample Generative Network for Developing Soft Sensors with Small Data. ACS Omega.

[50] Zhang L., Wei H., Lyu Z., Wei H., Li P. (2021). A Small-Sample Faulty Line Detection Method Based on Generative Adversarial Networks. Expert Syst. Appl..

[51] Shen L., Qian Q. (2022). A Virtual Sample Generation Algorithm Supporting Machine Learning with a Small-Sample Dataset: A Case Study for Rubber Materials. Comput. Mater. Sci..

[52] Liu X. (2004). Investigation on the Corrosion Behavior and Corrosion Prediction Model of Engineering Steels Used in Marine Environment. Ph.D. Thesis.

[53] Chen L., Su R.K.L. (2021). Corrosion Rate Measurement by Using Polarization Resistance Method for Microcell and Macrocell Corrosion: Theoretical Analysis and Experimental Work with Simulated Concrete Pore Solution. Constr. Build. Mater..

[54] Diao Y., Yan L., Gao K. (2021). Improvement of the Machine Learning-Based Corrosion Rate Prediction Model through the Optimization of Input Features. Mater. Des..

[55] WebElements The Periodic Table of the Elements. https://webelements.com.

[56] Roy A., Taufique M.F.N., Khakurel H., Devanathan R., Johnson D.D., Balasubramanian G. (2022). Machine-Learning-Guided Descriptor Selection for Predicting Corrosion Resistance in Multi-Principal Element Alloys. npj Mater. Degrad..

[57] Zhang Y., Wen C., Wang C., Antonov S., Xue D., Bai Y., Su Y. (2020). Phase Prediction in High Entropy Alloys with a Rational Selection of Materials Descriptors and Machine Learning Models. Acta Mater..

[58] Ji H., Wang H., Chen Q., Ma X., Cai Y. (2024). Corrosion Behavior Prediction for Hull Steels under Dynamic Marine Environments by Jointly Utilizing LSTM Network and PSO-RF Model. Ocean Eng..

[59] Lu Q., Liu S., Li W., Jin X. (2020). Combination of Thermodynamic Knowledge and Multilayer Feedforward Neural Networks for Accurate Prediction of MS Temperature in Steels. Mater. Des..

[60] Li L., Kumar Damarla S., Wang Y., Huang B. (2021). A Gaussian Mixture Model Based Virtual Sample Generation Approach for Small Datasets in Industrial Processes. Inf. Sci..

