# Supplementary material for: An Integrated Approach Using GA-XGBoost and GMM-RegGAN for Marine Corrosion Prediction Under Small Sample Size
Source: Materials (Basel). 2025 Aug 11;18(16):3760. doi: 10.3390/ma18163760 (PMC12387397; doi:10.3390/ma18163760)
Supplement: Supplementary file 1 [file materials-18-03760-s001.zip › materials-3785630-supplementary.pdf]

An Integrated Approach Using GA-XGBoost and GMM-RegGAN for Marine Corrosion Prediction Under Small Sample Size

Qian Chen [1], Yikun Cai [2], Yuqin Zhu [3,4], Haodi Ji [1], Xiaobing Ma [1,*] and Han Wang [1,*]

[1] School of Reliability and Systems Engineering, Beihang University, Beijing 100191, China
[2] School of Aeronautics and Astronautics, Sichuan University, Chengdu 610065, China
[3] Southwest Institute of Technology and Engineering, Chongqing 400039, China
[4] Ocean College, Zhejiang University, Hangzhou 316021, China
* Correspondence: maxiaobing@buaa.edu.cn (X.M.); wh216@buaa.edu.cn (H.W.)

Texts: 1

Tables: 5

Figures: 2

Pages: 8

Text S1:

In accordance with the feature engineering methodology outlined in Section 2.2, feature creation was performed on the marine steel corrosion dataset by transforming the original element features into physical, atomic, thermal, electronegativity, and orbital characteristics. Subsequently, a Pearson correlation analysis was conducted between the generated property features and the marine environmental features, with the results presented in Fig. S1. In the figure, the red rectangle illustrates the correlation matrix for the environmental features, the blue rectangle represents the correlation matrix for the property features derived through multiplication, and the green rectangle corresponds to the correlation matrix for the property features obtained via division.

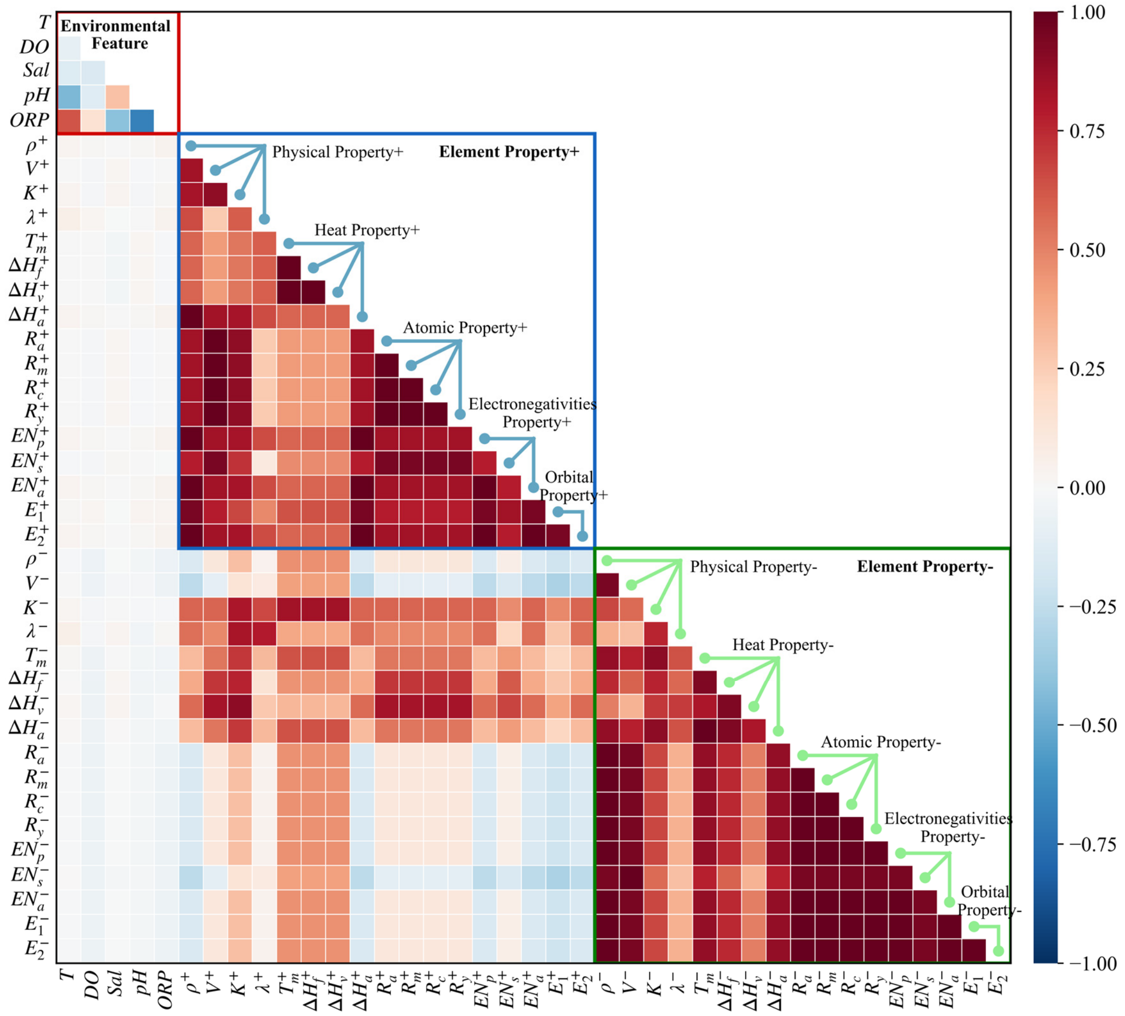

Figure S1. Results of Pearson correlation analysis.

As can be seen from the figure, the correlation coefficients between the environmental features and the property features are generally close to 0, indicating that there is minimal multi-

collinearity between the environmental and property features. This suggests that it is feasible to combine them for modeling the corrosion process of marine steel. The physical, thermal, atomic, electronegativity, and orbital property features exhibit strong correlations after being created through both multiplication and division methods. Within each property feature category, such as physical properties, the features exhibit a high correlation with one another. Additionally, there is also a noticeable high correlation between different property categories, such as atomic features, electronegativity features, and orbital features.

To mitigate the potential instability in model robustness caused by multi-collinearity among features, we initially employed the variance analysis and correlation analysis methods outlined in Section 2.3 for dimensionality reduction. A correlation coefficient threshold of 0.9 was applied during the correlation analysis. Based on the results of the variance analysis, the 39-dimensional feature set was reduced to 20 dimensions, as illustrated in Fig. S2.

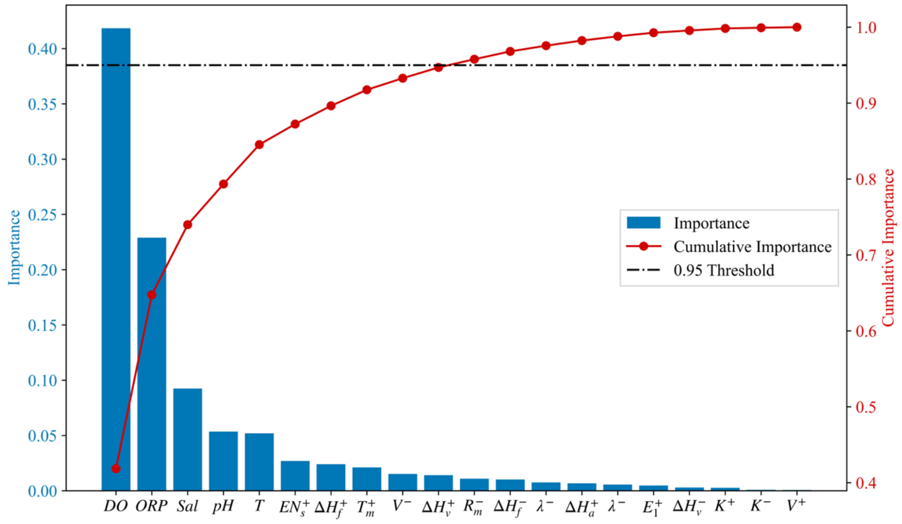

Figure S2. Result of feature importance and cumulative importance after correlation-based dimensionality reduction.

For the remaining 20-dimensional features, we further performed feature reduction based on feature importance analysis using GBDT, setting a cumulative importance threshold of 0.95. The results of the feature importance analysis and the feature selection based on cumulative feature importance are depicted in Fig. S2. The blue bars represent the feature importance of the 20-dimensional features after dimensionality reduction through correlation and variance analysis, the red line represents the cumulative feature importance, and the black dashed line represents the cumulative importance threshold. As shown in the figure, environmental features exhibit relatively

higher importance in the marine steel corrosion process, which may be attributed to the similarity in marine steel composition and the harshness of the marine corrosion environment. Additionally, other property features also have significant impacts on corrosion modeling. After the final dimensionality reduction, only five environmental features and six property features were retained, resulting in substantial improvement in both feature dimensionality and multi-collinearity issues.

Table S1 Experiment dataset of the six marine steels.

No.	3C						A3						16Mn					
	T	DO	Sal	pH	ORP	CR	T	DO	Sal	pH	ORP	CR	T	DO	Sal	pH	ORP	CR
1	25.90	6.71	30.10	5.10	378	16.40	24.27	0.80	32.56	8.10	171	2.55	27.36	5.04	32.17	7.86	310	10.15
2	29.35	6.09	29.00	6.30	400	16.90	27.45	2.60	35.37	7.96	287	10.96	28.72	6.68	32.21	8.00	325	11.90
3	27.90	6.18	31.50	7.00	363	15.57	27.23	4.20	31.94	7.89	289	12.00	28.45	9.90	31.95	7.93	309	14.77
4	24.00	7.95	30.20	8.10	324	13.65	28.72	6.80	32.21	8.00	325	13.33	24.27	0.80	32.56	8.10	171	2.34
5	28.00	5.05	31.40	9.20	240	13.24	28.52	8.40	32.10	8.01	345	17.31	23.95	7.61	9.17	8.04	231	7.83
6	27.32	3.21	29.31	8.20	281	12.91	28.45	9.90	31.95	7.93	309	22.48	24.73	6.06	17.33	7.88	322	7.47
7	27.87	6.55	31.68	7.20	356	14.06	23.95	7.61	9.17	8.04	231	8.13	24.60	7.52	24.42	7.57	227	9.58
8	28.27	6.98	28.20	6.60	384	15.47	24.95	6.80	16.29	7.82	341	9.07	24.51	7.02	32.00	8.16	308	11.12
9	30.70	7.15	31.74	6.50	401	16.28	24.60	7.52	24.42	7.57	210	10.74	23.88	6.45	41.67	7.83	237	8.76
10	29.37	6.82	30.12	6.20	414	17.11	27.32	3.21	29.31	8.20	281	13.59	27.32	3.21	29.31	8.20	281	8.53
11	24.27	0.80	32.56	8.10	171	3.61	24.00	7.95	30.20	8.10	324	12.89	24.00	7.95	30.20	8.10	324	8.64
12	27.45	2.60	35.37	7.96	287	7.94	27.78	6.35	31.38	7.20	356	13.61	27.86	6.40	24.83	7.00	356	8.94
13	27.23	4.20	31.94	7.89	289	9.63	27.97	6.05	31.94	6.60	384	14.60	28.00	6.53	27.30	6.60	384	9.12
14	27.48	5.90	32.39	7.83	331	10.58	30.70	7.15	31.74	6.50	401	15.00	30.70	7.15	31.74	6.50	401	11.09
15	28.75	6.80	32.22	8.00	340	11.43	29.37	6.82	30.12	6.20	414	15.39	29.37	6.82	30.12	6.20	414	12.00
16	28.52	8.40	32.10	8.01	345	12.52	25.90	6.71	30.10	5.10	378	18.22	25.80	6.30	30.10	5.10	368	16.10
17	28.45	9.90	31.95	7.93	309	22.64	29.35	6.09	29.00	6.30	400	16.45	29.35	6.09	29.00	6.30	400	10.54
18	23.95	7.61	9.17	8.04	231	10.94	27.00	6.70	30.70	7.00	350	12.60	27.00	6.31	30.50	7.00	350	8.50
19	24.73	6.06	17.33	7.88	321	11.45	27.90	5.15	31.50	9.20	264	9.08	9.50	4.26	32.31	8.20	95	8.93
20	24.60	7.52	24.42	7.57	210	11.83	25.55	6.67	31.00	8.09	320	12.49	28.00	5.26	31.30	9.20	252	7.27
21	24.51	7.02	32.00	8.16	308	12.55	24.31	6.42	40.67	7.88	250	8.75	17.74	7.23	34.50	7.97	167	11.11
22	23.65	6.51	41.34	7.67	245	8.40	24.11	6.38	41.00	7.98	228	8.99	20.23	6.66	33.41	8.04	201	12.79
23	16.74	7.11	33.55	8.25	178	10.85	17.45	7.48	34.08	8.10	135	17.05	25.85	5.99	32.72	8.08	292	11.52
24	21.11	6.03	33.44	8.03	295	11.45	21.95	8.28	34.64	7.95	113	17.34	31.18	5.27	33.75	8.07	113	11.10
25	25.57	6.70	32.19	8.09	325	11.87	27.19	4.91	33.50	7.99	275	15.48	-	-	-	-	-	-
26	31.16	4.38	33.21	7.94	242	8.92	-	-	-	-	-	-	-	-	-	-	-	-

No.	10MnPNbRe						10CrMoAl						16Mn					
	T	DO	Sal	pH	ORP	CR	T	DO	Sal	pH	ORP	CR	T	DO	Sal	pH	ORP	CR
1	25.86	1.70	33.97	8.03	229	4.31	25.86	1.70	33.97	8.03	229	7.86	25.86	1.50	33.97	8.03	229	4.51
2	27.23	4.18	31.94	7.89	289	6.80	27.36	5.04	32.17	7.86	310	10.46	27.23	4.18	31.94	7.89	289	9.39
3	28.60	6.50	32.12	7.89	346	8.54	28.72	6.68	32.21	8.00	325	12.32	28.72	6.68	32.21	8.00	325	11.47
4	28.45	9.90	31.95	7.93	309	12.76	28.45	9.90	31.95	7.93	309	18.28	28.52	8.40	32.10	8.01	345	14.96
5	24.31	9.23	32.57	8.03	191	24.59	26.18	9.15	33.89	8.07	241	21.50	28.45	9.90	31.95	7.93	309	22.03
6	24.73	6.06	17.33	6.06	322	7.40	24.73	6.06	17.33	7.88	322	5.38	23.95	7.61	9.17	8.04	231	7.48
7	25.10	7.28	22.71	7.76	227	8.35	25.10	7.28	22.71	7.76	227	13.38	24.95	6.80	16.29	7.82	341	11.01
8	25.85	6.52	31.00	8.01	277	11.74	24.81	6.87	30.00	8.08	265	14.59	25.59	7.04	21.00	7.95	244	12.75
9	24.24	6.40	41.45	7.91	242	7.03	24.24	6.40	41.45	7.91	242	6.66	24.81	6.87	30.00	8.08	265	16.55
10	27.32	3.21	29.31	8.20	281	15.39	9.50	4.26	32.31	8.20	95	7.79	24.96	6.32	40.00	8.08	254	7.99
11	17.10	6.51	33.93	8.14	189	10.59	27.32	3.21	29.31	8.20	281	8.92	27.32	3.21	29.31	8.20	281	11.09
12	28.19	6.35	29.66	7.00	356	16.46	17.74	7.45	34.48	8.00	149	9.97	16.47	7.11	33.56	8.25	178	10.85
13	28.21	6.18	31.96	6.60	384	18.01	28.32	6.25	31.96	7.20	356	10.20	28.26	6.30	31.62	7.20	356	12.31
14	30.70	7.15	31.74	6.50	401	18.32	27.93	6.14	31.21	6.60	384	11.06	27.86	6.45	31.40	6.60	384	12.38
15	29.37	6.82	30.12	6.20	414	18.24	30.70	7.15	31.74	6.50	401	11.39	30.70	7.15	31.74	6.50	401	13.74
16	25.90	6.71	30.10	5.10	378	17.10	29.37	6.82	30.12	6.20	414	12.94	29.37	6.82	30.12	6.20	414	14.23
17	29.35	6.09	29.00	6.30	400	19.07	25.90	6.71	30.10	5.10	378	16.91	25.90	6.71	30.10	5.10	378	20.95
18	27.40	6.60	31.80	7.00	343	17.79	29.35	6.09	29.00	6.30	400	11.24	29.35	6.09	29.00	6.30	400	17.39
19	22.00	7.95	30.30	8.10	324	14.43	28.20	6.41	31.70	7.10	350	7.05	28.30	6.07	31.60	7.10	352	11.16
20	28.30	4.60	31.40	9.20	250	15.10	18.70	8.40	30.04	8.10	324	6.21	24.00	7.95	30.10	8.10	324	8.52
21	21.40	5.92	33.44	8.02	204	12.27	28.00	5.15	31.20	9.20	244	6.36	28.00	4.99	31.40	9.20	246	8.07
22	26.11	6.10	32.63	8.06	308	9.73	21.09	6.93	33.85	7.99	173	12.90	20.29	6.58	33.42	8.01	209	14.73
23	31.39	4.26	33.51	7.96	151	4.49	25.57	6.70	32.19	8.09	325	8.82	25.57	6.70	32.19	8.09	325	16.81
24	25.86	1.70	33.97	8.03	229	4.31	-	-	-	-	-	-	31.12	3.85	32.92	7.89	208	7.81

Table S2 Hyperparameters of base models and the variation ranges.

Model	Hyperparameter	Domain definition
SVR	C	6.651
	gamma	0.842
	kernel function	poly
RF	n_estimators	103
	max_depth	8
	min_samples_split	4
LightGBM	n_estimators	47
	max_depth	4
	learning_rate	0.353
XGBoost	n_estimators	31
	max_depth	5
	learning_rate	0.292
ANN	hidden_layer	1
	nodes	124
	activation	logistic
	alpha	0.003

Table S3 Model RMSE for different numbers of virtual samples.

Num	MD-MTD	t-SNE	GMM	NITAE	CGAN	GMM-RegGAN
0	3.226	3.226	3.226	3.226	3.226	3.226
10	3.157	3.120	3.085	3.038	3.085	3.011
20	3.090	3.059	3.121	3.087	3.079	3.074
50	3.012	3.048	3.028	2.949	2.968	3.049
100	**2.961**	3.099	**2.998**	2.953	2.938	2.931
150	3.083	3.017	3.050	2.864	3.017	2.833
200	3.036	3.101	3.063	**2.855**	2.944	2.788
300	2.996	2.956	3.088	2.911	**2.933**	**2.744**
400	2.987	**2.926**	3.154	2.911	3.018	2.795
500	2.974	2.936	3.217	2.914	3.082	2.883

Table S4 Model MAE for different numbers of virtual samples.

Num	MD-MTD	t-SNE	GMM	NITAE	CGAN	GMM-RegGAN
0	2.483	2.483	2.483	2.483	2.483	2.483
10	2.498	2.460	2.435	2.382	2.434	2.334
20	2.447	2.389	2.424	2.391	2.438	2.357
50	2.346	2.378	2.372	2.332	**2.314**	2.356
100	**2.320**	2.382	**2.366**	2.319	2.333	2.238
150	2.419	2.376	2.404	**2.194**	2.364	2.182
200	2.364	2.447	2.398	2.230	2.342	2.127
300	2.358	2.344	2.385	2.283	2.355	**2.097**
400	2.342	**2.298**	2.429	2.293	2.405	2.128
500	2.325	2.307	2.478	2.270	2.453	2.154

Table S5 Model MAPE for different numbers of virtual samples.

Num	MD-MTD	t-SNE	GMM	NITAE	CGAN	GMM-RegGAN
0	0.269	0.269	0.269	0.269	0.269	0.269
10	0.266	0.263	0.261	0.254	0.256	0.251
20	0.261	0.255	0.258	0.258	0.257	0.255
50	0.253	0.255	**0.255**	0.257	0.244	0.256
100	0.253	0.257	0.257	0.255	**0.240**	0.241
150	0.257	0.257	0.262	**0.242**	0.246	0.236
200	0.253	0.265	0.259	0.251	0.242	**0.230**
300	0.254	0.250	0.265	0.260	0.242	0.231
400	0.252	0.247	0.271	0.261	0.251	0.237
500	**0.250**	**0.243**	0.277	0.259	0.260	0.244
